# Restoration of Polyamine Metabolic Patterns in* In Vivo* and* In Vitro* Model of Ischemic Stroke following Human Mesenchymal Stem Cell Treatment

**DOI:** 10.1155/2016/4612531

**Published:** 2016-06-16

**Authors:** Tae Hwan Shin, Geetika Phukan, Jeom Soon Shim, Duc-Toan Nguyen, Yongman Kim, Justin D. Oh-Lee, Hyeon-Seong Lee, Man Jeong Paik, Gwang Lee

**Affiliations:** ^1^Department of Molecular Science and Technology, Ajou University, Suwon 443-749, Republic of Korea; ^2^Department of Physiology and Department of Biomedical Sciences, Ajou University School of Medicine, Suwon 443-721, Republic of Korea; ^3^National Institute of Drug Quality Control, 48 Hai Ba Trung Street, Hanoi, Vietnam; ^4^Pharmicell Co., Ltd., Seongnam 462-806, Republic of Korea; ^5^Department of Psychology, Central Michigan University, Health Professions Building, MI 48859, USA; ^6^College of Pharmacy, Sunchon National University, Suncheon 540-950, Republic of Korea

## Abstract

We investigated changes in PA levels by the treatment of human bone-marrow-derived mesenchymal stem cells (hBM-MSCs) in ischemic stroke in rat brain model and in cultured neuronal SH-SY5Y cells exposed to oxygen-glucose deprivation (OGD). In ischemic rat model, transient middle cerebral artery occlusion (MCAo) was performed for 2 h, followed by intravenous transplantation of hBM-MSCs or phosphate-buffered saline (PBS) the day following MCAo. Metabolic profiling analysis of PAs was examined in brains from three groups: control rats, PBS-treated MCAo rats (MCAo), and hBM-MSCs-treated MCAo rats (MCAo + hBM-MSCs). In ischemic cell model, SH-SY5Y cells were exposed to OGD for 24 h, treated with hBM-MSCs (OGD + hBM-MSCs) prior to continued aerobic incubation, and then samples were collected after coculture for 72 h. In the* in vivo* MCAo ischemic model, levels of some PAs in brain samples of the MCAo and MCAo + hBM-MSCs groups were significantly different from those of the control group. In particular, putrescine, cadaverine, and spermidine in brain tissues of the MCAo + hBM-MSCs group were significantly reduced in comparison to those in the MCAo group. In the* in vitro* OGD system, *N* ^1^-acetylspermidine, spermidine, *N* ^1^-acetylspermine, and spermine in cells of the OGD + hBM-MSCs group were significantly reduced compared to those of OGD group.

## 1. Introduction

Bone-marrow-derived mesenchymal stem cells (BM-MSCs) are regarded as promising agents in ischemic stroke therapy [1–4] because of their differentiation plasticity, easy attainability, and weak immune response inducing ability [5–8]. In addition, mesenchymal stem cells are also known as suppressor of inflammation that is related to pathology of stroke [9, 10]. Accordingly, BM-MSCs have proven to be effective in ameliorating functional deficits as well as restoring tissues damages that occur after stroke [11, 12].

In previous clinical report, autologous human bone-marrow-derived mesenchymal stem cell (hBM-MSC) transplantation in patients with ischemic stroke suggested hBM-MSC's potential for providing functional recovery [13]. Although this study suggests that autologous hBM-MSC transplantation may be a safe treatment method for ischemic stroke, the precise underlying therapeutic mechanisms of hBM-MSC transplantation remain unknown. Furthermore, complex chemical, cellular, and physiological processes are involved in the dynamic regulation of metabolites during diseased states. Therefore, it is extremely difficult to instantaneously monitor and profile the dynamic metabolic changes in diseased state and therapeutic outcomes accurately. Among the diverse biogenic compounds with low-molecular weights occurring in metabolic pathways, polyamines (PAs) serve as the most important biochemical indicator for various pathological conditions [14–16]. The naturally occurring di-, tri-, and tetra-amines, polyamines (putrescine, cadaverine, spermidine, and spermine), which occur in metabolic pathways, are detected in high concentrations in the brain [17, 18]. PAs are secreted from intracellular compartments in several central nervous system (CNS) injuries, including focal cerebral ischemia in the ischemic cascade [18, 19]. Unregulated catabolism of PAs induces the production of harmful metabolites, such as hydrogen peroxide, by chelating Fe^2+^ via Fenton's reaction [20], thus increasing ischemic injury [21–23]. However, the precise characterization and the explicit role of PAs in brain ischemic conditions remain unknown.

In previous reports, spermine and spermidine were reported as free radical scavengers in rat brain homogenates, capable of reducing lipid peroxidation induced by prooxidant agents, including quinolinic acid (QA), iron (Fe^+2^), and sodium nitroprusside (SNP) [17, 24]. In particular, spermine reduced infarction and neurological deficit in a rat ischemic model as determined by using magnetic resonance imaging [25]. Even though the blood-brain barrier (BBB) strictly regulates the exchanges between brain and blood in normal metabolic conditions, induction of polyamine putrescine in cold-injury brains changes the integrity of focal BBB dysfunction [26]. These studies, taken together, support the suggestion that PAs and their metabolism play an important role as critical metabolomic components associated with cellular and chemical events during neurodegeneration following cerebral ischemia.

In order to further elucidate the beneficial or harmful role of altered PA levels as oxidant modulating agents in stroke, the present study was designed to analyse PA changes by evaluating treatment effects of hBM-MSCs in two different ischemic models. The first model utilized ischemic stroke condition in rats that had undergone middle cerebral artery occlusion (MCAo), while the second model employed oxygen-glucose deprivation (OGD) in cultured neuronal SH-SY5Y cells. In our previous report, regarding the rat stroke model, although there was no significant difference in infract volume and neurological status in MCAo induced groups irrespective of hBM-MSCs treatment before 7 days [27], the levels of free fatty acids (FFAs) in brain samples varied significantly in MCAo and hBM-MSC-transplanted MCAo groups in comparison to the control group at 5 days after MCAo. The study found that myristic, linoleic, and eicosenoic acids' levels of the hBM-MSC-transplanted MCAo group were significantly less than those of the control group [28]. Thus, we analysed polyamine metabolites at 5 days as this reflects the phenotype relatively well. In light of these observations, other metabolomic profiles, such as those related to PAs, may be linked with physiological, chemical, or cellular conditions in hBM-MSC transplantation in ischemic condition. Therefore, we extended our study to evaluate the metabolic patterns of PAs using both* in vivo* animal and* in vitro* cell models.

Although the therapeutic effects of stem cells were studied in ischemic animal models, the analysis of changes in PAs profiling associated with these conditions has not yet been attempted. Therefore, in the present study, simultaneous metabolic profiling analysis of aliphatic and acetylated PAs was performed to examine altered metabolic patterns of PAs in MCAo rat brains following intravenous hBM-MSCs injection and OGD SH-SY5Y cells treated with hBM-MSCs using coculture system. Outcomes from present experiments may provide new insight into our understanding of the complexity of biochemical and physiological events that occur in ischemic brain injury and after hBM-MSC replacement therapy in treatment for the stroke and other related disorders.

## 2. Results

### 2.1. Transplanted hBM-MSCs Were Detected in the Ischemic Border Zone

**Figure 1:**
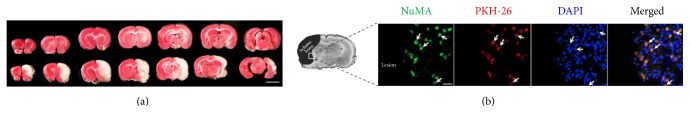
2,3,5-Triphenyltetrazolium chloride (TTC) staining and hBM-MSC staining with anti-NuMA antibody, PKH-26, and DAPI. (a) TTC-stained coronal brain sections of control (upper panel) and 5 days after MCAo (bottom panel). Scale bar = 5 mm. (b) hBM-MSCs labelled 5 days after MCAo. The localization of hBM-MSCs in the ischemic region was identified by NuMA (green), PKH-26 (red), and DAPI (blue); white arrows indicate double-positive cell. Scale bar = 20 *μ*m.

To assess the MCAo model, 2,3,5-triphenyltetrazolium chloride (TTC) staining was performed with ischemic rats. Infarcts, appearing as white areas, were mostly located within the ipsilateral cortex and striatum (Figure 1(a)). To trace transplanted hBM-MSCs* in vivo*, we assessed the fluorescence of cells labelled with PKH-26 in ischemic brain regions 4 days after injection. The ischemic region was identified by DAPI and hBM-MSCs labelled with NuMA were found in the margin (about 3–5% of 1 × 10^6^ hBM-MSCs, Figure 1(b)); these results are consistent with a previous report in the same model [27, 28]. However, we did not find NuMA and PKH-26 positive cells in the brain sections of control group and MCAo group. After confirmation of the stroke animal model and transplantation of hBM-MSCs in the ischemic brain region, we analysed the metabolic patterns of PAs four days after the transplantation of hBM-MSCs into the MCAo model.

### 2.2. PAs Levels in the Brain of Rat Model

To investigate the change of PA levels between control, control + hBM-MSCs, MCAo, and MCAo + hBM-MSCs (1 × 10^6^) groups, brain PA profiling analysis was performed. Nine PAs were detected in rat brain tissue with great variations in levels being observed within each group (Table 1). Compared to the control, the levels of putrescine, cadaverine, *N* ^1^-acetylspermidine, and spermidine were significantly increased in the MCAo group, while the levels of putrescine and spermidine were significantly increased in the MCAo + hBM-MSCs group. However, there was no significant difference in polyamine levels between control and control + hBM-MSCs, except for spermidine increment. In addition, putrescine, cadaverine, and spermidine were significantly reduced in the MCAo + hBM-MSCs group in comparison to the MCAo group, but still they were of higher levels compared to control group.

**Figure 2:**
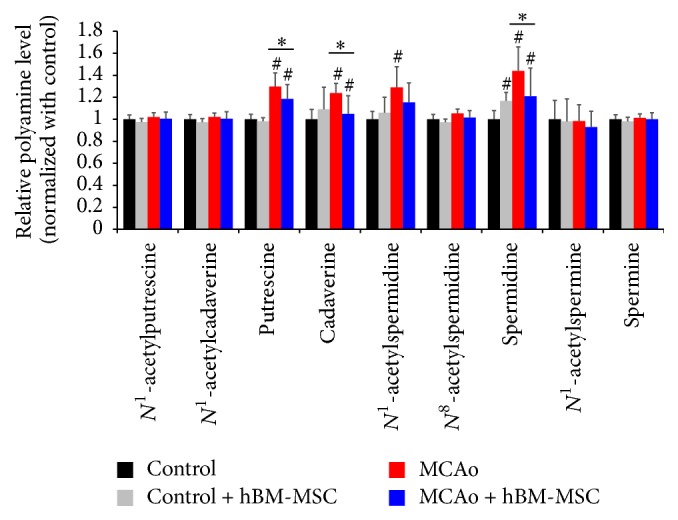
Bar plot of MCAo and MCAo + hBM-MSCs groups based on mean levels of the nine polyamines in rat brain tissues as variables after normalization to the corresponding mean values of the control group. ^#^ *p* < 0.05 (comparison with control) and ^*∗*^ *p* < 0.05 (comparison between MCAo and MCAo + hBM-MSCs groups). Data shown are means ± SE for three independent experiments.

**Figure 3:**
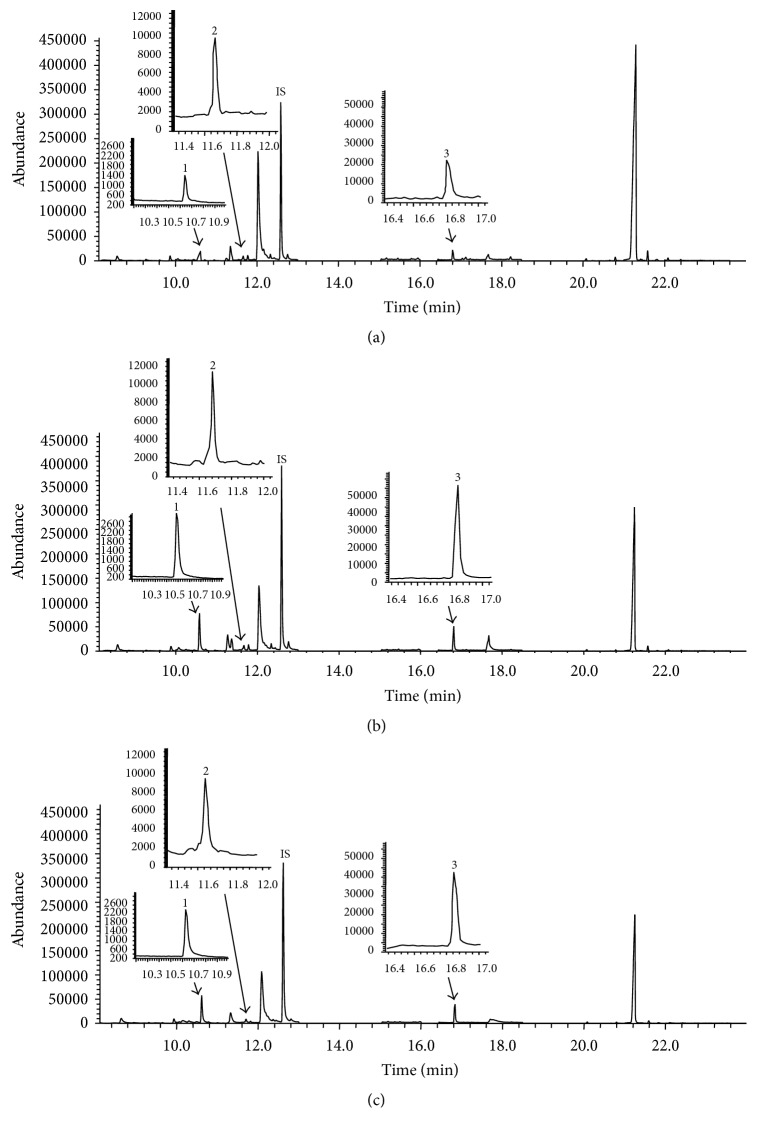
GC-SIM-MS chromatograms of putrescine (1), cadaverine (2), and spermidine (3) in rat brain tissues from control (a), MCAo (b), and MCAo + hBM-MSCs groups (c). IS: internal standard (1,6-diaminohexane).

The levels of each of the nine PAs in brain tissues of the MCAo and MCAo + hBM-MSCs groups were normalized to the corresponding mean values from the control group (Figure 2). PA levels of the MCAo and MCAo + hBM-MSCs groups display multiple control mean values ranging from 0.9 to 1.4. Compared to the control group, four and three PAs were observed as having significant variation in the MCAo and MCAo + hBM-MSCs groups, respectively. In both the MCAo and MCAo + hBM-MSCs groups, the level of spermidine was found to be the most altered, followed by putrescine in comparison to the control group. Interestingly, the PAs, which were elevated in MCAo, were reduced in the MCAo + hBM-MSCs group, with the level of most PAs approximating the level of the control group. Representative SIM chromatograms also revealed that putrescine, cadaverine, and spermidine of the MCAo + hBM-MSCs group were significantly lower than those of the MCAo group, but still they were of higher levels compared to control group (Figure 3).

### 2.3. PAs Levels in Cells under OGD

To mimic the MCAo animal model, we used Boyden chamber (Transwell) to assess whether coculture with hBM-MSCs exhibited the same effects to OGD SH-SY5Y system as an* in vitro* ischemic model. PA profiling analysis was performed with the control (*n* = 3), OGD (*n* = 3), and OGD + hBM-MSCs (*n* = 3) groups. Seven PAs were detected in the cells of each group (Table 2). The levels of putrescine, *N* ^1^-acetylspermidine, *N* ^8^-acetylspermidine, spermidine, *N* ^1^-acetylspermine, and spermine were significantly elevated in the OGD group compared to the control group. In particular, *N* ^1^-acetylspermine and spermine were increased by more than 2-fold. Compared to the control group, the levels of putrescine, *N* ^1^-acetylspermidine, spermidine, *N* ^1^-acetylspermine, and spermine in the OGD + hBM-MSCs group were significantly increased, whereas *N* ^8^-acetylspermine was significantly reduced. Compared to the OGD group, the level of putrescine was significantly increased in the OGD + hBM-MSCs group, whereas the levels of *N* ^1^-acetylspermidine, spermidine, *N* ^1^-acetylspermine, and spermine were significantly decreased.

**Figure 4:**
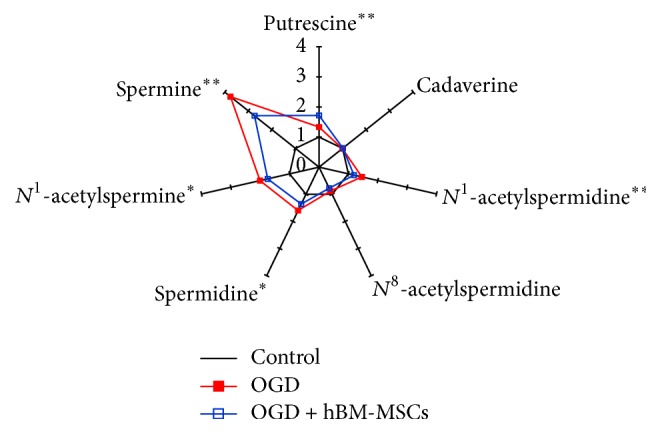
Star symbol plots of OGD and OGD + hBM-MSCs groups based on the mean levels of the seven polyamines in cultured cells for 72 h as the variables after normalization to the corresponding control group mean values. ^*∗*^ *p* < 0.05 and ^*∗∗*^ *p* < 0.01 (comparison between OGD and OGD + hBM-MSCs groups). Data shown are means ± SE for three independent experiments.

**Figure 5:**
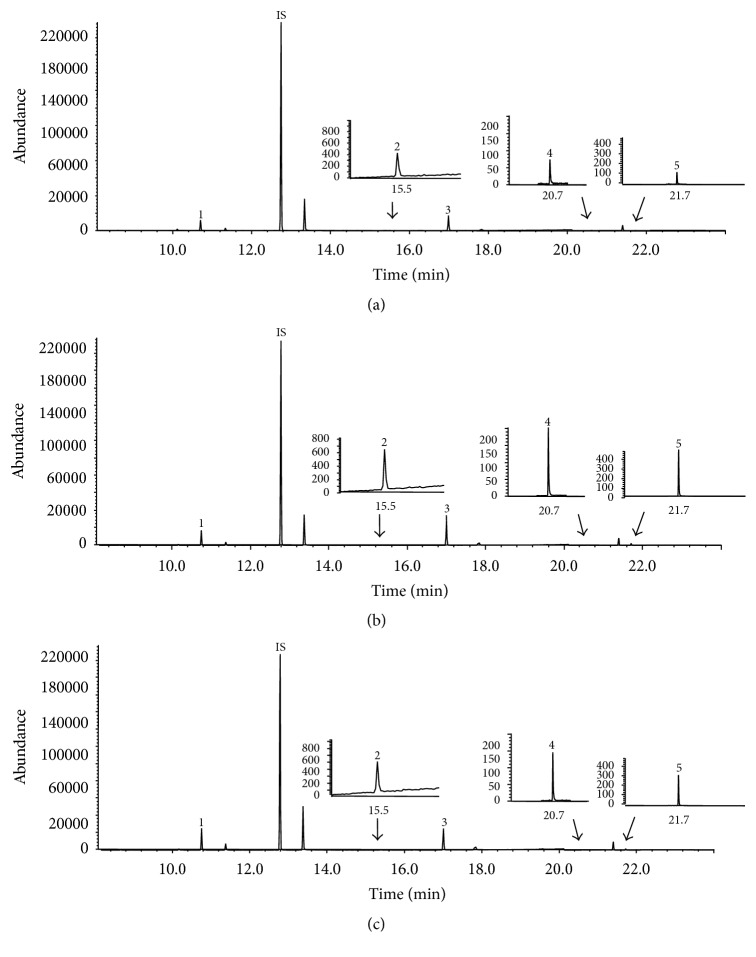
GC-SIM-MS chromatograms of putrescine (1), *N* ^1^-acetylspermidine (2), spermidine (3), *N* ^1^-acetylspermine (4), and spermine (5) in cells from control (a), OGD (b), and cocultured OGD cells (c) with hBM-MSCs for 72 h. IS: internal standard (1,6-diaminohexane).

Similar to the procedures used in the MCAo animal model, each level of the seven PAs in cells of the OGD and OGD + hBM-MSCs groups was normalized to the corresponding mean values from the control group. These normalized values were then utilized for star graphs composed of seven rays, so that the differences in the mean values among the control, OGD, and OGD + hBM-MSCs groups were exhibited clearly (Figure 4). PA levels from the OGD and OGD + hBM-MSCs groups were elevated manifold (ranging from 0.8 to 3.8), compared to the control mean values. In the OGD and OGD + hBM-MSCs groups, spermine was the most changed followed by *N* ^1^-acetylspermine, compared to the control group. Interestingly, the PAs, which were elevated in OGD, except for putrescine, were reduced in the OGD + hBM-MSCs group, similar to the* in vivo* results. The representative SIM chromatograms also revealed that putrescine and spermine of the OGD + hBM-MSCs group were considerably altered compared to the OGD group (Figure 5).

## 3. Discussion

To the best of our knowledge, the present study is the first demonstration of altered PA metabolism in the brains of ischemic rats and OGD cells following transplantation of hBM-MSCs. Despite the complexity of the generated results, the present findings provided evidence that hBM-MSCs transplantation could ameliorate PA metabolic dysfunctions associated with ischemic cellular injuries in both brain tissues and OGD cells.

In the ischemic stroke rat model, compared to the control group, the levels of 4 of 9 PAs were increased in brains of rats in the MCAo group. All PAs that were increased in the MCAo group, except for *N* ^1^-acetylspermidine, were considerably reduced in the MCAo + hBM-MSCs group (Table 1). In the OGD cell model, the levels of 5 of 7 PAs, except for cadaverine and *N* ^8^-acetylspermine, were increased in oxygen-glucose deprived cells compared to the control group. However, all elevated PAs in the OGD group, except for putrescine, were noticeably reduced in the OGD + hBM-MSCs group (Table 2). These results collectively suggested that the disturbance of PA metabolism by MCAo- and OGD-induced ischemic condition could be restored to a near-normal state after transplantation of hBM-MSCs.

In the present study, hBM-MSCs transplantation showed limited restoration of cellular polyamine homeostasis in rat MCAo models because complete restoration to basal level depends on various internal and external factors. Moreover, polyamine metabolism is a reversible process in which various polyamines alter between intermediate forms [29], which makes quantification of polyamines in cells difficult. This also contributes to limited interpretation of our data regarding polyamine alterations in the OGD model. In order to accurately quantify polyamines in OGD model, tracing of polyamines by radiolabelling their precursor ornithine will be required.

In the ischemic animal model, changes in the metabolite profile of spermidine were observed in the MCAo and MCAo + hBM-MSCs groups. This supported the possibility that MCAo might induce the elevation of spermidine through the putrescine metabolic pathway, such as by the activation of spermidine synthase (SpdS) due to ischemic conditions [30]. Also, spermine and spermidine concentrations were reported to increase in response to acute hypoxia in fetal rat brains that coincided with cell differentiation and growth, suggesting a protective role in hypoxic brain [31]. Since large physiological changes in putrescine and cadaverine have been described in brain ischemia, abnormally high levels of these metabolites observed in the present MCAo group could contribute to the further imbalance in other PAs [17]. In addition, upregulation of both spermidine and putrescine as well as their acetylated form suggests an increase in biosynthesis as well as in the interconversion pathway. Our results provided evidence that transplantation of hBM-MSCs in ischemic brain was capable of restoring metabolic disturbances caused by ischemia and further that prevention of ischemic stroke might be related, in part, to normalization of the release of PAs from the intracellular compartment in the ischemic brain.

Stem cells are responsible for cell renewal and maintenance of tissue homeostasis [32]. Increasing evidences indicate that hBM-MSCs promote functional recovery in animal model of ischemic stroke. We reported that upregulation of the endogenous recovery mechanism at the peri-infarct area (neurogenesis) has an important role of hBM-MSCs in functional recovery after ischemic stroke after 14 days [27]. In addition, transplantation of hBM-MSCs restored free fatty acid composition in ischemic stroke rat model [28]. In this study, we postulate that transplantation of hBM-MSCs contribute to maintenance of metabolic homeostasis before or during early pathological condition in ischemic stroke rat model.

In a previous study using a similar ischemic MCAo stroke model, both spermine and spermidine levels were reported to be significantly decreased at 6 and 24 h after MCAo in the ischemic cortex compared to the control cortex [33]. On the other hand, current findings showed that the level of spermidine was increased, whereas the level of spermine remained unaltered in MCAo condition when compared to the control condition. These dissimilar results may be attributed, at least in part, to differences in methodological approaches used in preparing brain tissue. We used total brain, obtained from brain tissues 5 days after MCAo. Although blood volume contributes only about 1% to total tissue polyamines [34], the animals in the present study were perfused with physiological saline prior to brain tissue collection in order to exclude the possibility of the effect of blood on PA expression. In addition, the spermine in brain tissues of the control group was higher than spermidine, which showed a different pattern in comparison to previous report [35]. This may be explained by our use of the whole brain rather than particular regions of the brain.

In MCAo ischemic rat model, it is important to note that the altered PAs in the ischemic rat brain were measured at only one time point after hBM-MSC transplantation, namely, 4 days after the hBM-MSC transplantation. PA change in brains was also observed at only one time point after ischemia, namely, 5 days after MCAo. Future studies, therefore, should evaluate other postischemic, as well as posttransplant, time points. This may be important for more accurate assessment of the alterations in PA metabolite profiles in a time-dependent manner, allowing the analysis of the impact of ischemia* per se*, as well as of its interaction with hBM-MSC transplantation, as a function of stem cell survival, migration, and functional integration into the host CNS tissue. In addition, other factors, including stem cell dose and its route of delivery, should be investigated in relation to specific biochemical processes involving PA metabolism in order to achieve optimal treatment condition and to validate the potential clinical utility of hBM-MSC stem cell therapy in ischemic conditions such as stroke and related disease states.

Furthermore, in an OGD coculture system, PA levels were also measured at only one time point, namely, at 72 h after coculture with hBM-MSCs, since, in this case, there were no earlier cytoprotective effects of hMSC. In fact, at 24 h after exposure to OGD, viability of SH-SY5Y cells was about 80% or less in the absence of hBM-MSCs treatment and remained at about the same level after hBM-MSC treatment (data not shown). Therefore, future OGD cell experiments should include analyses using various OGD exposure time and/or varying hBM-MSCs concentrations to better delineate the functional relationship between hBM-MSCs therapeutic effects and altered PA metabolomics in this model. It is also of significant importance to recognize that the present* in vitro* OGD experiments utilized the SH-SY5Y human neuroblastoma cell line, a well-characterized and well-established cell model system for studying neuronal growth* in vitro*. Therapeutic effects of stem cells in ischemic rats, however, are not directly related to neuroblastoma cells but instead are more closely related to other types of cells, such as cortical neurons, microglia, and astrocytes [36, 37]. Future* in vitro* studies concerning the effects of hMSC in ischemic conditions should incorporate the use of these cell lines as well as primary cells which evaluate polyamine effects on receptors (i.e., NMDA) associated with synaptic functions [38]. However, glial cells are immune cells of the brain and assessing the effect of OGD and hBM-MSCs transplantation in these cells and neurons, complex multilevel investigation such as 3D culture is required [39]. hBM-MSCs also induce microgliosis and astrogliosis in transplanted brain [40]. Moreover, secondary paracrine effects of hBM-MSCs on resident microglia and neurons further complicate the hypoxic microenvironment. Such a study will require more careful consideration of several critical factors. Accordingly, the present* in vitro* data and its potential clinical implications should be interpreted cautiously until further work with additional cell lines is completed.

The changes of PA levels from the* in vitro* model are very different from those obtained in ischemic rat model. Particularly, spermine concentration is modified in the presence of OGD and then after hBM-MSCs cocultures. This discrepancy is considered to be responsible for selective neuronal cell line* in vitro* model and presence of several cell types in brain tissues such as neurons, microglia, and astrocytes. Further research is clearly warranted to delineate precise underlying restorative mechanisms of hBM-MSCs transplantation in ischemia, since the involvement of PAs in the ischemic damage and related alterations are still unknown.

## 4. Materials and Methods

### 4.1. Transient MCAo Animal Model

Ischemic stroke was introduced by the intraluminal suture occlusion model in a stroke rat model. Adult male Sprague-Dawley rats weighing 250–300 g were anesthetized, by the use of face mask, with 4% isoflurane, and maintained with 1.5% isoflurane in 30% O_2_ and 70% N_2_O. During the surgery, rectal temperature was maintained at 37.0–37.5°C with heating pads. Transient MCAo using a method of intraluminal vascular occlusion modified in our laboratory was used [28]. Briefly, a 4-0 surgical monofilament nylon suture with rounded tip was introduced through the common carotid artery (CCA) and used to block the lumen of the internal carotid artery (ICA). Two hours after MCAo, reperfusion was performed by withdrawal of the suture until the tip cleared the lumen of CCA. Surgical procedures for sham groups were identical to those used for MCAo surgery, except for omission of the suture insertion. The use of animals was approved by the Animal Care and Use Committee of Ajou University Hospital.

### 4.2. 2,3,5-Triphenyltetrazolium Chloride (TTC) Staining and Immunohistochemistry

To confirm the establishment of ischemic animal model, rats were anesthetized with chloral hydrate 24 hours after MCAo. Brains were then removed and immediately sectioned coronally into six slices (each 2 mm in thickness) in a rodent brain matrix (Harvard Instrument, South Natick, MA), as described previously [28]. Briefly, brain slices were placed in 2% triphenyltetrazolium chloride (TTC; T-8887; Sigma-Aldrich, St. Louis, MO), incubated at 37°C for 40 min and fixed by immersion in 10% formalin. Using a flatbed color scanner, the stained sections were photographed and scanned for further analysis. The infarcts were mostly located within the ipsilateral cortex and the striatum in three representative slices (Figure 1(a)). For immunohistochemistry, animals were sacrificed one day after MCAo. Brains were fixed by transcardial perfusion with saline, followed by perfusion and immersion in 4% paraformaldehyde. The brains were kept overnight in paraformaldehyde at 4°C and then embedded in a 30% sucrose solution until they sank. Coronal sections 30 *μ*m in thickness were cut using a model CM1800 cryostat (Leica, Wetzlar, Germany). The sections were washed three times with phosphate-buffered saline (PBS), nonspecific binding was blocked with 10% horse serum, and each section was stained with 1 : 500 dilution of mouse monoclonal human nuclei matrix antigen (NuMA; Calbiochem, San Diego, CA). Thereafter, sections were washed and incubated with secondary antibody. To assess the number of hBM-MSCs within the transplant, the total number of NuMA-positive cells in the forebrain (bregma-1, approximately 1 mm) was calculated on ten sequential slides at intervals of 150 *μ*m, and the number of NuMA-positive cells was counted by summing those found on all 10 slides as described previously [27, 28]. To trace transplanted hBM-MSCs* in vivo*, the cells were labelled using a PKH 26 Red Fluorescence Cell Linker Kit (Sigma, St. Louis, MO, USA) according to the manufacturer's instructions. Briefly, brains were harvested from MCAo or sham-operated animals and fixed overnight with 4% paraformaldehyde in 0.1 M phosphate buffer. Frozen brains were sectioned with a Cryocut-microtome system (Leica, Germany). Sections were then incubated with 4,6-diamidino-2-phenylindole (DAPI; Fluka, USA) for 15 min at room temperature to counterstain the nuclei and mounted.

### 4.3. Animals and Experimental Groups

One day after MCAo, animals were randomly divided into three groups as in our previous report [28]: (1) sham ischemia + phosphate-buffered saline (PBS) injection (control, *n* = 10), (2) MCAo + PBS injection (*n* = 6), and (3) MCAo + hBM-MSCs (1 × 10^6^, *n* = 7) injection. All animals were allowed to survive for 5 days after MCAo. MCAo + hBM-MSCs group showed functional recovery with the adhesive-removal test and modified neurological severity score (mNSS) test compared to the MCAo + PBS group, at 14 days after MCAo [27]. However, we could not detect any functional recovery at 5 days in both groups.

Tissue extracts, from brain harvested for analysis, were prepared by homogenization with DEPC water (Sigma, St. Louis, MO). Brain samples were freshly obtained under fasting conditions and were immediately stored at −70°C until analysed. To exclude the possibility of variation in diet and other factors, we used rats that were fed the same diet and maintained in the same housing environment throughout the study period.

### 4.4. hBM-MSCs Culture

hBM-MSCs were provided from Pharmicell (Seoul, Korea) under culture in GMP (Good Manufacturing Practice) conditions as in our previous report [13, 28]. Briefly, cells were incubated at 37°C in 5% CO_2_ in flasks for one day and nonadherent cells were removed by replacement of the medium. Once the cells reached about 80% confluence, they were harvested with 0.05% trypsin and 0.53 mmol/L EDTA (GIBCO, Grand Island, NY) for 5 minutes at 37°C, replated in a flask, cultured again for 3–5 days, and harvested. In order to achieve a sufficient dose, hBM-MSCs used in these experiments were collected from six passages [27].

### 4.5. Chemicals and Reagents

Putrescine, cadaverine, spermidine, spermine, *N* ^1^-acetylputrescine, *N* ^1^-acetylcadaverine, *N* ^1^-acetylspermidine, *N* ^8^-acetylspermidine, *N* ^1^-acetylspermine, 1,6-diaminohexane, ethylchloroformate (ECF), and pentafluoropropionyl anhydride (PFPA) were obtained from Sigma-Aldrich. Diethyl ether, ethyl acetate, and dichloromethane of pesticide grade were obtained from Kanto (Tokyo, Japan). Sodium chloride, obtained from Junsei (Tokyo, Japan), was washed successively with methanol, acetone, dichloromethane, and diethyl ether, followed by drying under a vacuum (100°C, 1 h). Sodium hydroxide was obtained from Duksan (Seoul, South Korea). All other chemicals were of analytical grade.

### 4.6. Sample Preparation of Brain Tissues

Sample preparation for assay of PAs in rat brain samples was performed according to our previous method [41] for the following brain samples: control, control + hBM-MSCs, MCAo, and MCAo + hBM-MSCs groups. Brain tissue in 5 mL distilled water was homogenized (3 min, 30,000 rpm) in an ice water bath using a model Pro 200 rotor/stator-type tissue homogenizer (Pro Scientific, Oxford, CT). An aliquot equivalent to 20 mg of brain tissue, including 1,6-diaminohexane (0.2 *μ*g), as the internal standard (IS) of the homogenate, was vortex-mixed with 1 mL acetonitrile for 3 min. The mixture was centrifuged at 15,000 rpm and 15 min for protein precipitation. Briefly, ethoxycarbonyl (EOC) reaction was performed in aqueous phase by 10 min vortex with 20 *μ*L ECF present in 1 mL of the dichloromethane phase. Then, the mixture was saturated with sodium chloride and sequentially extracted with diethyl ether (3 mL) and ethyl acetate (2 mL). The extracts were evaporated under a nitrogen gentle stream at 40°C, which were converted as PFP derivative (60°C for 30 min) with PFPA (20 *μ*L) for analysis by gas chromatography-mass spectrometry (GC-MS) in the SIM mode (GC-SIM-MS) as described previously [41].

### 4.7. Sample Preparation of Cells

PA profiling analysis was performed, as previously described [41], in the following cell groups: control, oxygen-glucose deprivation (OGD), and OGD + hBM-MSCs groups. IS (0.1 *μ*g) was added to each cell aliquot (4 × 10^5^ cells) after freeze-thaw lysis, which were subjected to the aforementioned EOC-PFP reactions. The reaction mixtures were then analysed by GC-SIM-MS as described previously [41].

### 4.8. Exposure of SH-SY5Y Human Neuroblastoma Cells to Oxygen-Glucose Deprivation

SH-SY5Y cells (passage < ~50) were cultured in an aerobic incubator with 5% CO_2_ and humidified at 37°C. High glucose Dulbecco's Modified Eagle Medium (DMEM), containing 10% fetal bovine serum (FBS) and 1% penicillin/streptomycin (P/S), was used for culturing. Oxygen-glucose deprivation was performed as described previously [42]. Briefly, the culture medium was replaced by glucose-free DMEM containing 10% FBS, and SH-SY5Y cells were adapted by incubation in an aerobic incubator for 4 h at 8 × 10^4^ cells per cm^2^. OGD cells were then transferred to an anaerobic chamber, containing a gas mixture of 95% N_2_ and 5% CO_2_, and humidified at 37°C. The medium was subsequently replaced by glucose-free DMEM that had been purged using N_2_. After 24 h OGD incubation, OGD was terminated by removing the cultures from the chamber and replacing the medium with glucose-enriched DMEM. Other SH-SY5Y cells exposed to the OGD condition were also treated with 10^5^ cells per cm^2^ hBM-MSCs in the insert well using transwell for 72 h (OGD + hBM-MSCs). The OGD group without hBM-MSCs consisted of culturing aerobically for the same period of time. All samples were collected 72 h later.

### 4.9. GC-MS

As outlined in our previous report, GC-MS analysis in SIM mode for quantitative analysis of PAs in rat brain tissue was conducted with an Agilent 6890N gas chromatograph interfaced to an Agilent 5975 mass-selective detector (70 eV, electron impact mode) and installed with an Ultra-2 (SE-54 bonded phase; 25 m × 0.20 mm I.D., 0.11 *μ*m film thickness) cross-linked capillary column (Agilent Technologies, Santa Clara, CA) [28].

### 4.10. Star Symbol Plotting

PA values were measured in cells. For each sample, PA values were normalized to the corresponding mean values in the normal group. Each normalized value was then plotted as a line radiating from a common central point. The far ends of seven lines for cells were joined together to produce a star pattern for each group using the MS Excel program, as described in the previous report [43, 44].

## 5. Conclusion

Outcomes of our metabolomic experiments have provided new insight into our understanding of the complexity of biochemical and physiological events that occur in ischemic brain injury and the therapeutic effects of hBM-MSCs in treatment of stroke. More importantly, elucidation of PA metabolism after hBM-MSCs transplantation could guide the future development of effective measures or therapeutic strategies for stem cell therapy for protection against cerebral ischemia or reduction of ischemic injury.

## Figures and Tables

**Table 1:** Polyamine levels in brain tissues from rats in the control group, group with transient MCAo, and the MCAo group following transplantation with hBM-MSCs.

Number	Compound	Polyamine levels (nmol, mean ± SD) in rat brain tissue (g)				
		Control group (*n* = 10)	Control + hBM-MSCs group (*n* = 7)	MCAo group (*n* = 7)	MCAo + hBM-MSCs group (*n* = 7)	*p* value^d^
1	*N* ^1^-Acetylputrescine	374.7 ± 15.3	365.6 ± 12.0 (0.1)^a^	382.3 ± 22.8 (0.2)^b^	376.6 ± 15.0 (0.4)^c^	0.3
2	*N* ^1^-Acetylcadaverine	419.2 ± 17.7	408.3 ± 13.8 (0.1)	428.1 ± 26.9 (0.2)	421.1 ± 14.7 (0.4)	0.3
3	Putrescine	109.3 ± 5.0	107.4 ± 3.6 (0.2)	141.9 ± 14.2 (0.000003)	129.7 ± 13.6 (0.0003)	0.06
4	Cadaverine	59.5 ± 5.3	64.9 ± 11.9 (0.1)	73.7 ± 9.8 (0.0008)	62.4 ± 5.2 (0.1)	0.01
5	*N* ^1^-Acetylspermidine	137.0 ± 9.9	145.1 ± 19.3 (0.1)	176.6 ± 24.4 (0.0002)	158.0 ± 25.7 (0.02)	0.1
6	*N* ^8^-Acetylspermidine	231.8 ± 10.4	225.6 ± 6.9 (0.1)	244.2 ± 14.6 (0.03)	235.5 ± 9.3 (0.2)	0.1
7	Spermidine	172.6 ± 13.8	201.4 ± 13.4 (0.0003)	248.4 ± 44.3 (0.00006)	208.6 ± 37.6 (0.007)	0.05
8	*N* ^1^-Acetylspermine	140.7 ± 24.2	138.2 ± 28.8 (0.4)	138.4 ± 20.5 (0.4)	130.7 ± 21.2 (0.2)	0.3
9	Spermine	513.6 ± 21.3	503.5 ± 19.4 (0.2)	520.7 ± 30.4 (0.3)	513.6 ± 18.4 (0.5)	0.3

^a^Student's *t*-test at 95% confidence level on the mean values of control and control + hBM-MSCs groups.

^b^Student's *t*-test at 95% confidence level on the mean values of control and MCAo groups.

^c^Student's *t*-test at 95% confidence level on the mean values of control and MCAo + hBM-MSCs groups.

^d^Student's *t*-test at 95% confidence level on the mean values of MCAo and MCAo + hBM-MSCs groups.

**Table 2:** Polyamine levels in cells from control, OGD, and OGD + hBM-MSCs groups for 72 h.

Number	Compound	Polyamine levels (ng, mean ± SD) in cells (4 × 10^5^)			
		Control group (*n* = 3)	OGD group (*n* = 3)	OGD + hBM-MSCs group (*n* = 3)	*p* value^c^
1	*N* ^1^-Acetylputrescine	ND^d^	ND	ND	
2	*N* ^1^-Acetylcadaverine	ND	ND	ND	
3	Putrescine	16.4 ± 1.5	21.8 ± 0.9 (0.002)^a^	28.1 ± 0.9 (0.0003)^b^	0.001
4	Cadaverine	12.5 ± 0.2	12.4 ± 0.4 (0.794)	12.6 ± 0.3 (0.861)	0.501
5	*N* ^1^-Acetylspermidine	45.6 ± 1.8	66.5 ± 0.8 (0.0003)	54.3 ± 4.7 (0.026)	0.006
6	*N* ^8^-Acetylspermidine	64.4 ± 0.6	56.7 ± 4.0 (0.047)	50.1 ± 3.3 (0.03)	0.082
7	Spermidine	132.5 ± 3.0	212.1 ± 16.7 (0.0002)	180.4 ± 6.9 (0.004)	0.024
8	*N* ^1^-Acetylspermine	63.4 ± 6.0	127.3 ± 9.7 (0.0005)	110.6 ± 0.6 (0.0005)	0.050
9	Spermine	64.9 ± 6.2	243.8 ± 15.1 (0.0002)	177.6 ± 9.5 (0.0003)	0.001

^a^One-way ANOVA, *p* value < 0.05 between control and OGD groups.

^b^One-way ANOVA, *p* value < 0.05 between control and OGD + hBM-MSCs groups.

^c^One-way ANOVA, *p* value < 0.05 between OGD and OGD + hBM-MSCs groups.

^d^Not determined.
